# Corrigendum: Synthetic torpor triggers a regulated mechanism in the rat brain, favoring the reversibility of Tau protein hyperphosphorylation

**DOI:** 10.3389/fphys.2023.1256251

**Published:** 2023-07-25

**Authors:** Fabio Squarcio, Timna Hitrec, Emiliana Piscitiello, Matteo Cerri, Catia Giovannini, Davide Martelli, Alessandra Occhinegro, Ludovico Taddei, Domenico Tupone, Roberto Amici, Marco Luppi

**Affiliations:** ^1^ Department of Biomedical and Neuromotor Sciences, University of Bologna, Bologna, Italy; ^2^ Centre for Applied Biomedical Research—CRBA, St. Orsola Hospital, University of Bologna, Bologna, Italy; ^3^ Department of Experimental, Diagnostic and Specialty Medicines, University of Bologna, Bologna, Italy; ^4^ Department of Neurological Surgery, Oregon Health and Science University, Portland, OR, United States

**Keywords:** deep hypothermia, microtubules, melatonin, glycogen synthase kinase 3β, hippocampus, parietal cortex

In the published article, there was an error in Figure 4E as published. The only **histogram bars** represented in **Panel E left side** (referred to P-Cx [i.e., Parietal cortex]) are wrong, together with the relative “y” scale. However, the representative Western blot bands depicted at the bottom of the histograms are correct, as described in the original caption that is also reported below. The corrected Figure 4 and its correct original caption appear below.

**FIGURE 4:**
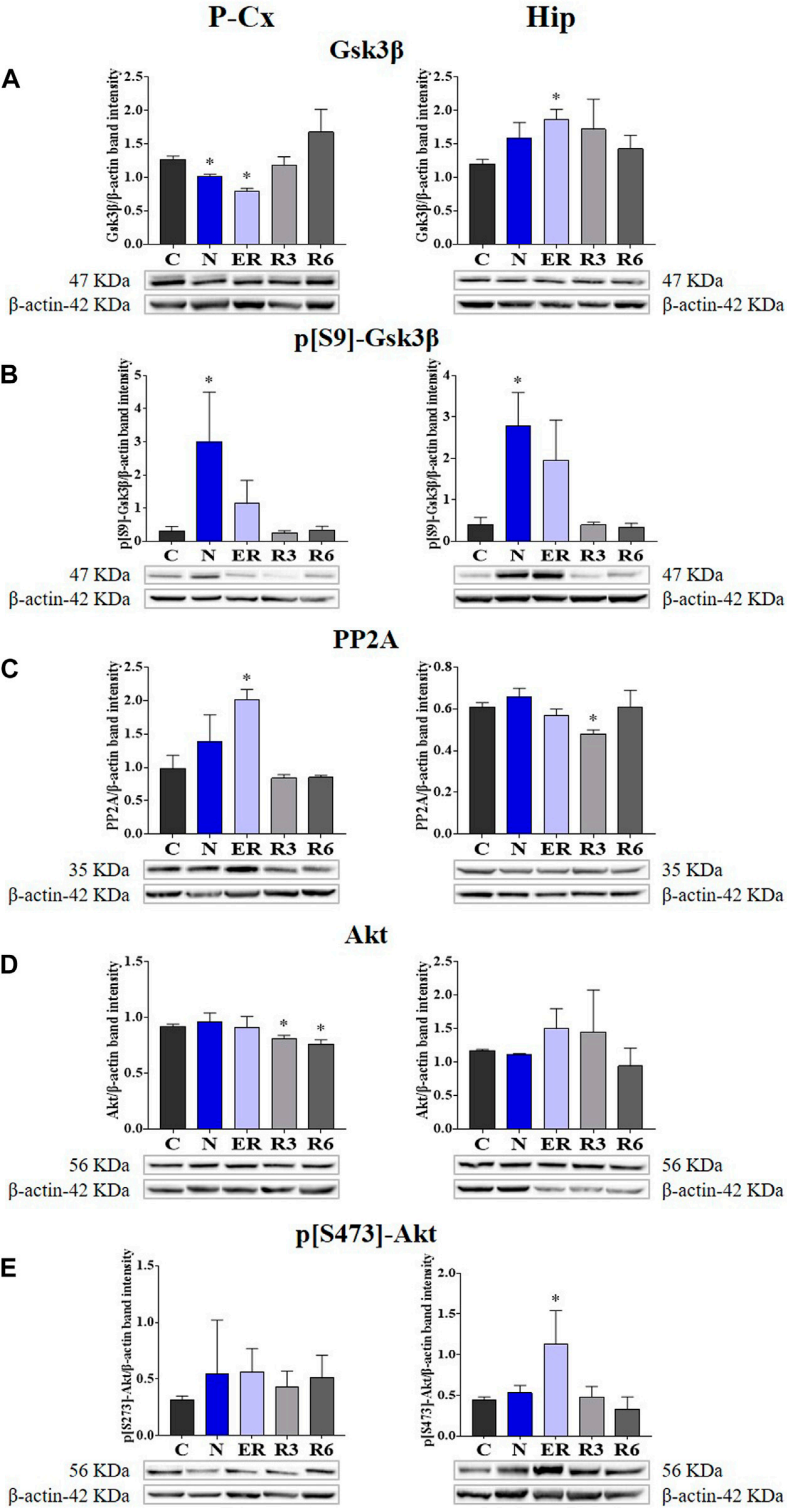
Western blot detection of the main enzymes involved in phosphorylation and dephosphorylation of Tau, determined in brain extracts of the parietal cortex (P-Cx) and hippocampus (Hip). Below each histogram, WB representative samples are shown for each experimental condition. **(A)** glycogen-synthase kinase-3β (GSK3β), the main kinase targeting Tau; **(B)** p[S9]-GSK3β (inactive form of GSK3β, phosphorylated at Ser9); **(C)** protein phosphatase-2A (PP2A), the main phosphatase targeting Tau; **(D)** different isoforms of Akt (protein kinase-B; Akt 1/2/3), kinases targeting GSK3β at Ser9 and antiapoptotic factors; **(E)** p[S473] Akt, the active form of Akt 1/2/3, phosphorylated at Ser473. Data are normalized by β-actin and expressed as means ± S.E.M., *n* = 3. *: *p* < 0.05 vs. C. Experimental groups (see **Figure 1**): C, control; N, samples taken at nadir of hypothermia, during ST; ER, early recovery, samples taken when Tb reached 35.5°C following ST; R3, samples taken 3 h after ER; R6, samples taken 6 h after ER.

The authors apologize for these errors and state that this does not change the scientific conclusions of the article in any way. The original article has been updated.

