# Supplementary material for: Synthetic torpor triggers a regulated mechanism in the rat brain, favoring the reversibility of Tau protein hyperphosphorylation
Source: Front Physiol. 2023 Mar 9;14:1129278. doi: 10.3389/fphys.2023.1129278 (PMC10034179; doi:10.3389/fphys.2023.1129278)
Supplement: Supplementary file 1 [file DataSheet1.PDF]

Supplementary Material

Synthetic torpor triggers a regulated mechanism in the rat brain, favoring the reversibility of Tau protein hyperphosphorylation.

Fabio Squarcio[1†], Timna Hitrec[1†], Emiliana Piscitiello[1,4], Matteo Cerri[1], Catia Giovannini[3,4], Davide Martelli[1], Alessandra Occhinegro[1,4], Ludovico Taddei[1], Domenico Tupone[1,2], Roberto Amici[1] and Marco Luppi[1,4*]

[1]Department of Biomedical and NeuroMotor Sciences, University of Bologna, Bologna, Italy.

[2]Department of Neurological Surgery; Oregon Health & Science University; Portland, OR – USA.

[3]Department of Experimental, Diagnostic and Specialty Medicine, University of Bologna, Bologna, Italy.

[4]Centre for Applied Biomedical Research – CRBA, University of Bologna, St. Orsola Hospital, Bologna, Italy.

†These authors contributed equally to this work and share first authorship

*** Correspondence:**
Dr. Marco Luppi, Department of Biomedical and NeuroMotor Sciences, University of Bologna, Bologna, Italy. Piazza di Porta san Donato, 2 – 40126 Bologna, Italy. Phone number: +390512091731. E-mail: marco.luppi@unibo.it

1.1 Supplementary Figures

C

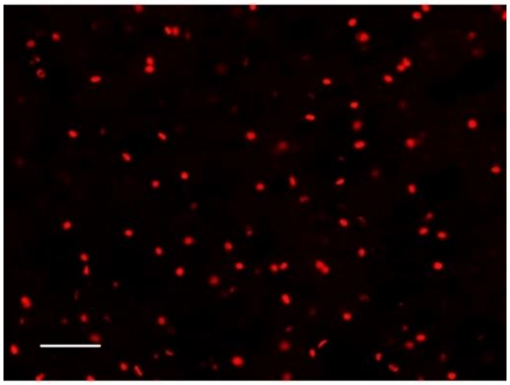

N

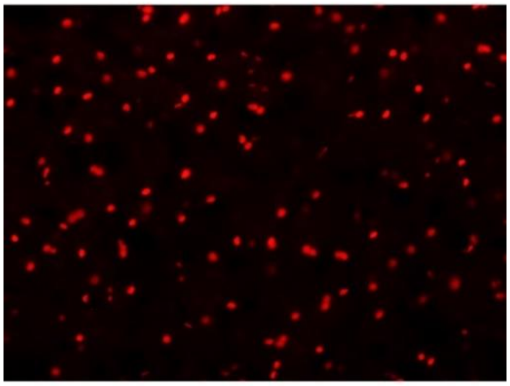

R3

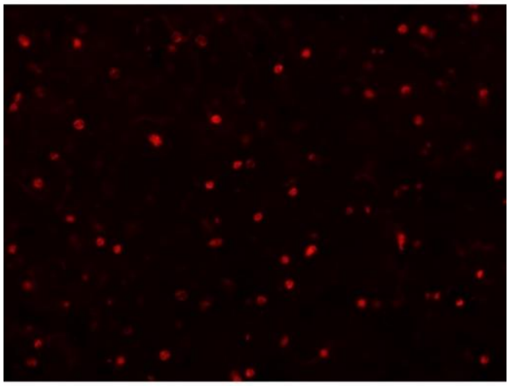

R6

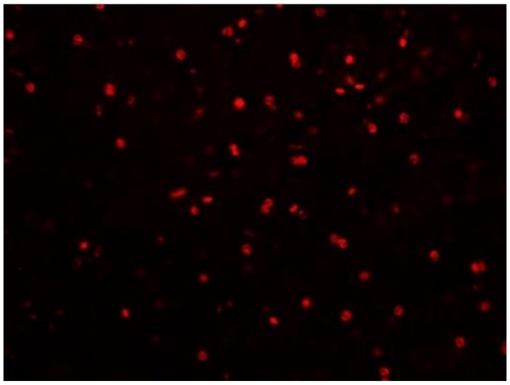

Supplementary Figure 1. cleaved-Caspase 3 in P-Cx.

Representative pictures showing samples from the parietal cortex (P-Cx) stained for cleaved-Caspase-3 (secondary antibody conjugated with Alexa-594). Experimental groups (see Figure 1): C, control; N, samples taken at nadir of hypothermia, during synthetic torpor (ST); R3, samples taken 3h after returning to euthermia; R6, samples taken 6h after returning to euthermia. Calibration bar: 50 μm.
